# First evidence of *Besnoitia bennetti* infection (Protozoa: Apicomplexa) in donkeys (*Equus asinus*) in Belgium

**DOI:** 10.1186/s13071-018-2993-3

**Published:** 2018-07-18

**Authors:** Emmanuel Liénard, Adriana Nabuco, Sophie Vandenabeele, Bertrand Losson, Irène Tosi, Émilie Bouhsira, Françoise Prévot, Shukri Sharif, Michel Franc, Caroline Vanvinckenroye, Yannick Caron

**Affiliations:** 1 Laboratoire de Parasitologie et Maladies Parasitaires, ENVT, Université de Toulouse, Toulouse, France; 2 IHAP, INRA, ENVT, Université de Toulouse, Toulouse, France; 3 Waterloo, Belgium; 4 University of Ghent, Faculty of Veterinary Medicine, Merelbeke, Belgium; 5 University of Liège, Faculty of Veterinary Medicine, Liège, Belgium

**Keywords:** Scleral and labial cysts, *Besnoitia bennetti*, Donkey, Besnoitiosis, Europe

## Abstract

**Background:**

Besnoitiosis is caused by different species of intracellular protozoan parasites belonging to the family Sarcocystidae and affecting multiple host species worldwide. Including *B. besnoiti*, ten species are described infecting animals. Among ungulates, *Besnoitia bennetti* infects horses, donkeys and zebras and was described in Africa and in the USA where donkey besnoitiosis is considered as an emerging disease.

**Case presentation:**

A two-year-old male donkey was purchased in May 2016 in poor body condition (cachexia, alopetic areas and pruritus mainly on neck and head) by the present owner in Le Roeulx (Belgium) from a milk producing donkey farm in Frasnes-lez-Buissenal (Belgium). Shortly after its purchase and shearing, the donkey presented with crusts, hyperkeratosis (both flanks and neck) anorexia and cachexia. A treatment with phoxim was given with no improvement. A cutaneous biopsy of hyperkeratotic skin was performed in July. It showed a perivascular eosinophilic infiltrate with a large thick walled cyst located in the dermis containing numerous bradyzoites. This was highly suggestive of besnoitiosis. Several skin biopsy samples were obtained for qPCR analysis and confirmed the presence of *Besnoitia* spp. DNA. Further laboratory diagnosis tests were performed (western blot and rDNA sequencing) confirming *Besnoitia bennetti* aetiology for the male. For the female, the punch-biopsy, haematology and qPCR were negatives but the western blot showed the presence of antibodies directed to *Besnoitia* spp. Further clinical examination performed in August highlighted scleral pinhead sized cysts (pearl) in the right eye and between nares. Another ten-year-old female donkey purchased in France and sharing the same accommodation showed a good clinical condition, but a thorough clinical examination showed the presence of numerous cysts on the inner face of upper labial mucosa. A daily treatment based on sulfamethaxzole and trimethoprim (Emdotrim 60% Mix®, 30 mg/kg) was given orally and some improvement was noticed.

**Conclusion:**

This is the first evidence of *Besnoitia bennetti* infection (Protozoa: Apicomplexa) in donkeys (*Equus asinus*) in Belgium.

**Electronic supplementary material:**

The online version of this article (10.1186/s13071-018-2993-3) contains supplementary material, which is available to authorized users.

## Background

Besnoitiosis is caused by different species of intracellular protozoan parasites belonging to the family Sarcocystidae (phylum Apicomplexa) affecting multiple host species worldwide. The cyst-forming parasite, *Besnoitia besnoiti*, in cattle has received more attention since cattle besnoitiosis was recognized in 2010 as re-emerging in Europe by the European Food Safety Authority (EFSA) [1]. Its propagation from historic endemic areas in Europe (Spain, Portugal and southern France) took place during the last two decades and currently at least eight European countries have reported clinical cases in imported or autochthonous animals: Switzerland, Italy, Germany, Croatia, Greece, Hungary, Belgium and Ireland [2–6]. For instance, a western blot revealed infection in a six-year-old Blonde d’Aquitaine bull which was imported in Belgium in 2012 from the Pyrenees Mountain endemic region in southern France [7]. The first stage of infection is the acute systemic phase (tachyzoites are present in the blood) characterized by fever, lymphadenopathy and oedematous swelling, sometimes associated with mortality, abortion and male transient or definitive sterility [8]. The second and chronic stage is the scleroderma phase or stage characterized by the development of numerous tissue cysts filled with bradyzoites in the dermis, vaginal and tracheal mucosa and on the ocular sclera, the latter localization being pathognomonic. These cysts are responsible for skin lesions with hyperkeratosis and alopecia [4].

Including *B. besnoiti*, ten species are described, infecting four and six species of large or small animals, respectively [2]. Among ungulates, *Besnoitia bennetti* infects horses, donkeys and zebras and was described prior to 1992 in Africa (Sudan and South Africa) [2, 9–12] and in the USA [13–15] where donkey besnoitiosis is considered as an emerging disease [16, 17]. The most striking clinical sign shared with cattle besnoitiosis is the development of multifocal pinpoint parasitic cysts in the skin, between the nares, on the internal and external pinnae, limbs and perineum and along the limbal margin of the sclera. Moreover, as observed in *B. besnoiti* infection, many infected animals remain clinically healthy [18]. Attempts to identify the definitive host for *B. bennetti* have been unsuccessful, precluding researchers from elucidating the parasite’s life-cycle in equine infection [18].

In this report, we describe two cases of besnoitiosis in donkeys, for the first time in Belgium, using a full set of diagnostic tools.

## Case presentation

### Consultation purpose

In July 2016, a private veterinarian referred a donkey characterised by poor body condition and chronic skin lesions to the Faculty of Veterinary Medicine of Liège (Belgium).

### Clinical history

**Fig. 1:**
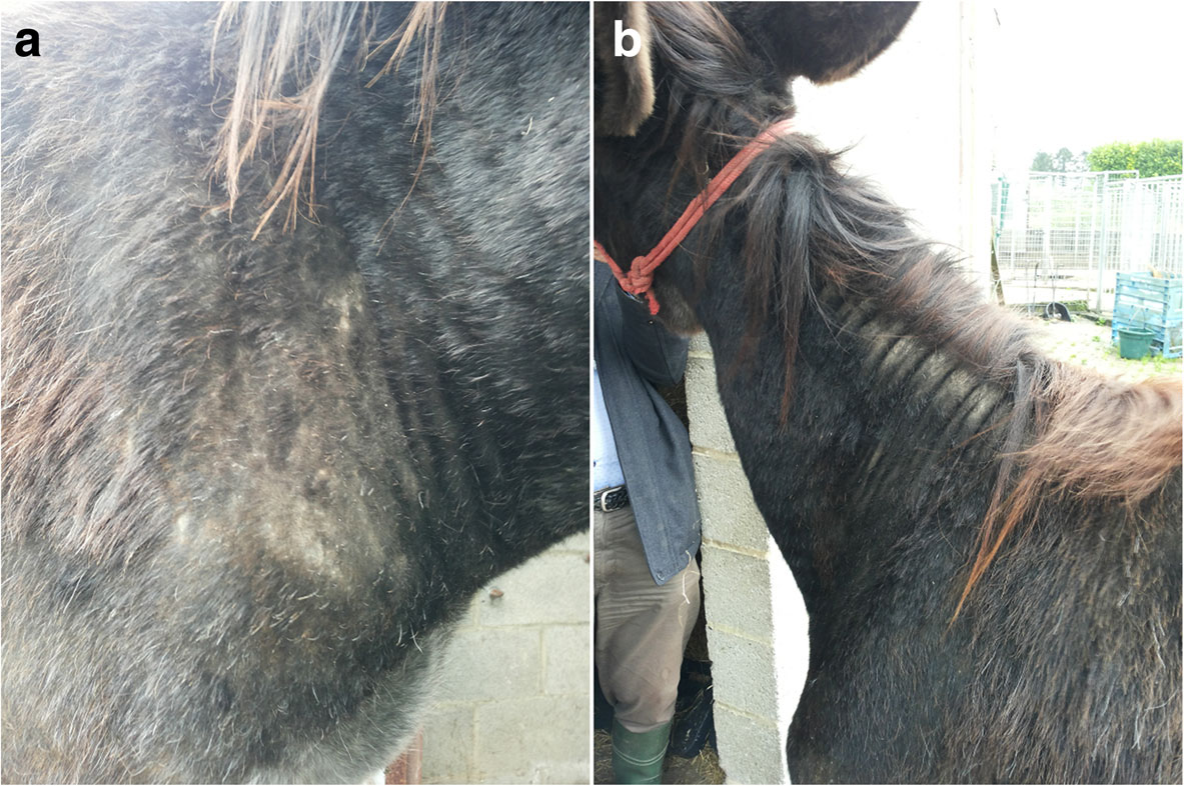
Patient’s skin lesions: **a** alopetic and crusty area on the right shoulder, **b** hyperkeratosis (neck)

A two year old male donkey (Grand Noir du Berry breed), was purchased in May 2016 in poor body condition (weight loss, alopecic areas, pruritus mainly on neck and head, dirty long and matted hair) by the present owner in Le Roeulx in Belgium (50°31'49.48"N, 4°06'56.33"E). This jack came from a milk producing donkey farm in Frasnes-lez-Buissenal, Belgium (50°40'11.31"N, 3°37'11.19"E; Fig. 1). A treatment with phoxim (Sarnacuran®) was given with no improvement.

### Clinical descriptions

**Fig. 2:**
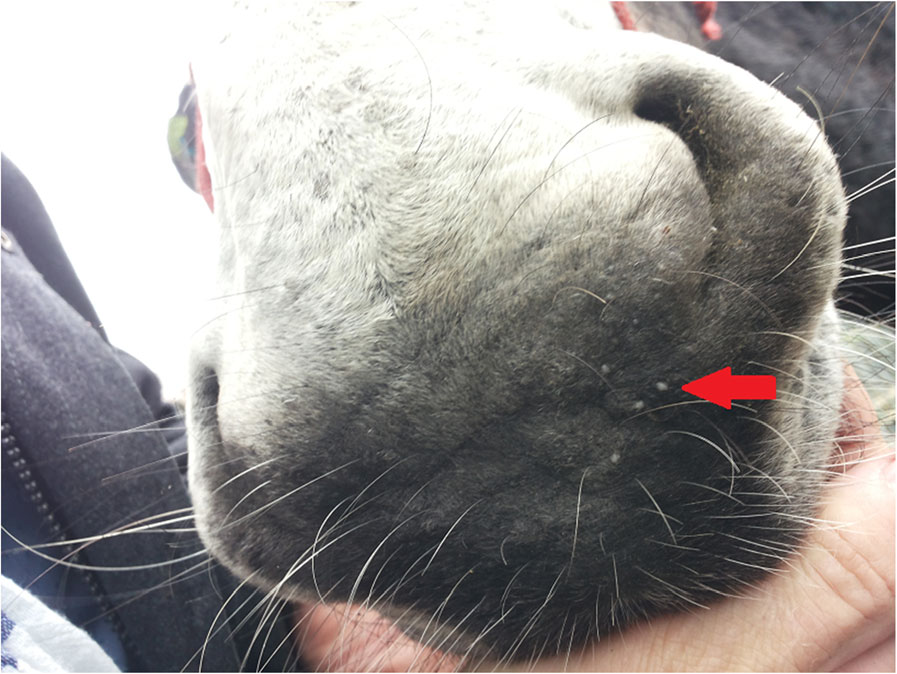
Scleral pin-head sized cysts (pearl; red arrow) between nares

Shortly after its purchase, the animal was shorn revealing crusts and hyperkeratosis (on both flanks and the neck). The animal was anorexic and in poor body condition. A closer clinical examination in August highlighted scleral pinhead sized cysts (pearl) in the right eye and between nares (Fig. 2). The rest of the examination was unremarkable.

Another ten year old female donkey (Grand noir du Berry breed), purchased several years ago in France (Loire region) by the same owner and sharing the same accommodation, was in good clinical condition. However, further clinical examination showed the presence of numerous cysts on the inner face of upper labial mucosa.

The two donkeys were kept in a fenced area (below 1 ha). The animals were fed a standard donkey food regimen composed of hay, supplemented with protein-containing grain (oats) and occasional fruits and vegetables. Previous medical treatments included routine vaccinations and prophylactic deworming (based on ivermectin). Other animal species (cats, dogs, chickens and rodents) had free access to the farm and the paddock buildings. Numerous flies were observed in the paddocks (*Stomoxys calcitrans* and *Musca* spp.).

### Laboratory examination, diagnosis and prognosis

#### Skin scraping

Skin scrapings were performed in different places (mainly flanks and neck where the lesions were the most obvious) on both animals. A culture on Sabouraud dextrose agar-chloramphenicol (0.5 per 1000 m/w for three weeks at 37°C) yielded a negative result for ringworm. Microscopic examination of the hair revealed neither fungal spores nor ectoparasites. A Giemsa staining of an impression smear also gave negative results.

#### Histopathology

Several skin biopsies were taken using an 8 mm biopsy punch. Histopathology and DNA extraction for qPCR and PCR were performed on those skin samples. After collection of the sample, the material was stored in 10% phosphate-buffered formalin for histological examination and in 70% ethanol for molecular analyses. The formalin-fixed sample was bisected and embedded in paraffin wax at 56 °C, sectioned at 4 μm, cut and stained with haematoxylin and eosin for routine evaluation. This technique was performed by a veterinary diagnostic laboratory (Medvet, Antwerpen, Belgium).

**Fig. 3:**
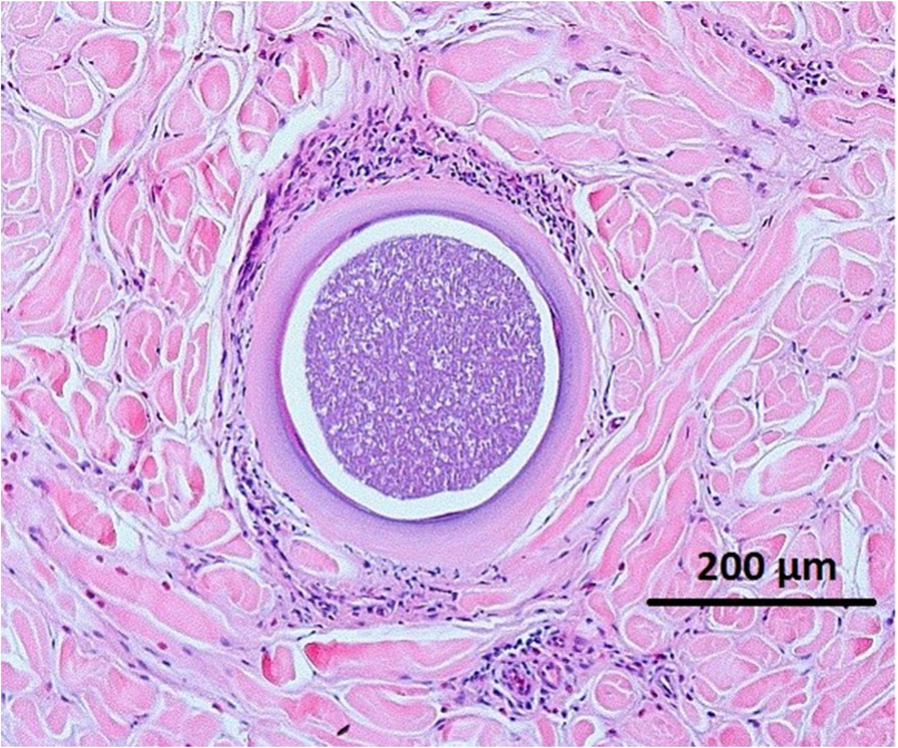
Histopathological preparation from the male donkey showing a cyst full of bradyzoites (haematoxylin eosin; 10×)

In the clinically affected patient, focal spongiosis epidermis was observed. In the dermis, there was a perivascular eosinophilic infiltrate and in the deep dermis a large thick-walled (28 μm) cyst (313 × 344 μm) packed with bradyzoites present (Fig. 3). With the exception of a perivascular eosinophilic infiltrate, the histopathological preparation from the other animal was unremarkable.

#### Blood sampling and analysis

Several blood samples were taken for haematology and biochemistry analysis from both patients. A blood sample from the male gave unremarkable results except a light anaemia [haemoglobin 10.1 d/l (normal range 11–19 d/l), red blood cells 5.22 10^6^/mm^3^ (normal range 6.5–12.5 10^6^/mm^3^), haematocrit 30% (normal range 32–52%)], an eosinophilia [15.5%; 1631/mm^3^ (normal range < 5% and < 500/mm^3^, respectively)] and an increased gamma globulin fraction [32.8%; 23.9 g/l (normal range 13–21% and 5.5–19 g/l, respectively)]. A blood sample from the female revealed only an eosinophilia (12.7%, 916/mm^3^).

**Fig. 4:**
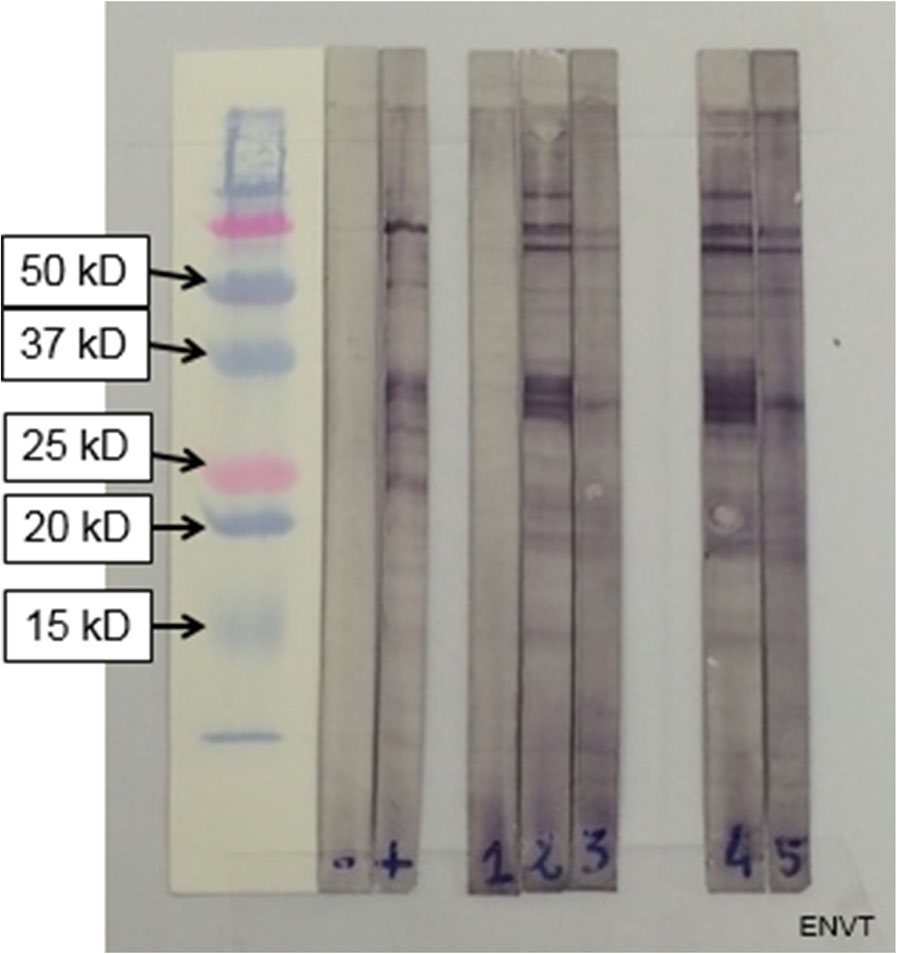
Western blot results. Lane -: sera from uninfected cattle; Lane +: sera from infected cattle; Lane 1: sera from uninfected donkey; Lane 2: male serum (4/8/2016); Lane 3: female serum (4/8/2016); Lane 4: male serum (9/9/2016); Lane 5: female serum (9/9/2016)

An in-house western blot was performed to detect specific antibodies in both donkeys. The procedures were adapted from previous publications [19]. Sera were tested from two sampling dates (4th August 2016 and 9th September 2016) at 1:50 dilution for each donkey. Peroxidase-labelled goat anti-horse IgG conjugate [anti-horse IgG (Whole molecule)-peroxidase, Sigma-Aldricht, Saint-Quentin Fallavier, France] was used at 1:150 dilution. A molecular weight marker was used (Precision Plus Protein standards, Bio-Rad, Basel, Switzerland). Serum from a cow chronically infected with *B. besnoiti* was used as a positive control, whereas serum samples from uninfected cattle and donkeys were used as negative controls. Serum was considered positive when at least four out of ten bands of specific tachyzoites antigens (45, 40, 37, 34, 30, 27, 22, 17, 16 and 15 kDa) were observed [5, 16]. Figure 4 shows the results of the western blot. Serum from the male donkey exhibited a strong positive result according to criteria defined by others [20] and was even stronger one month later; the female donkey yielded a similar observation.

#### qPCR

DNA from skin biopsies (50 mg) from both donkeys were extracted with the QIAmp® DNA Mini Kit (Qiagen, Courtaboeuf, France) according to the manufacturer’s recommendations. *Besnoitia* spp. internal transcribed spacer 1 (ITS-1) amplification was performed with the commercial PCR kit AdiaVet^TM^ Besnoitia (AES Chemunex, Bruz, France). The quantitative PCR was performed with the Stratagene MX3005P thermal cycler (Agilent Technologies, La Jolla, CA, USA), and results were analysed using the MxPro QPCR version 4.10 software (Agilent Technologies). A threshold cycle (Ct) value equal to or greater than 40 corresponded to a negative result [20]. Sterile water and genomic DNA from healthy donkeys were used as negative controls. A positive control was provided by a genomic DNA extract of purified *B. besnoiti* tachyzoites cultivated on Vero cells [21]. The skin sample from the male was highly positive with a Ct of 25 but no *Besnoitia* DNA was detected in skin sample from the female.

#### Species identification using partial rDNA sequencing

**Fig. 5:**
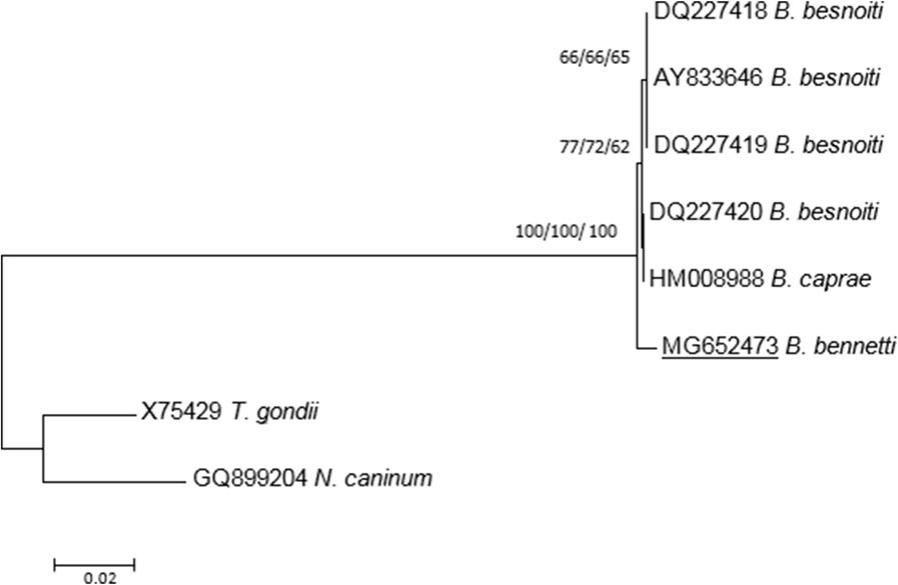
Phylogenetic tree based on 938 bp rDNA sequence (containing complete sequences of *5.8S* gene and ITS1 and ITS2 fragments and partial sequences of *18S* and *28S* genes) reconstructed using NJ/MP/ML methods showing the position of the isolated Belgian strain (GenBank: MG652473) and other *Besnoitia* from cattle and goat deposited in the GenBank database. Bootstrap support (1000 replicates) is shown at each node as NJ/MP/ML; bootstrap values below 60% are not shown. The tree is rooted with *T. gondii* (GenBank: X75429) and *N. caninum* (GenBank: GQ899204). The scale-bar indicates the number of substitutions per site

Following qPCR results, only the positive *Besnoitia* DNA sample was used for specific identification. A portion of ribosomal DNA cistron containing 5 genes [*18S*, ITS1, *5.8S*, internal transcribed spacer 2 (ITS2) and *28S*] was amplified using the primers Bes-F and Bes-R as designed by others [7] and synthesized by Eurogentec (Angers, France). The PCR reaction was performed in a total volume of 50 μl per sample with 5 μl DNA template, 10 pmol of each primer and 25 μl of the Taq PCR Master Mix® (Qiagen) containing (final concentrations) 1.5 mM MgCl_2_, 200 μM of each dNTP, 1.25 units of Taq polymerase and Qiagen PCR Buffer 1× (pH = 8.7). The PCR was performed in a GeneTouch Thermal Cycler (Bioer, Hangzhou, China) using the following program: 94 °C for 3 min (initial denaturation), 35 cycles at 94 °C for 45 s, annealing at 61 °C for 60 s and extension at 68 °C for 60 s, with a final extension step at 68 °C for 10 min. Positive and negative controls were the same as those used for qPCR. PCR products were sequenced directly in both directions with the primers used for DNA amplification at the genomic facility GeT-Purpan (Federative Institute for Bio-medical research, Toulouse, France). The 938 bp rDNA sequence containing complete sequences of *5.8S* and internal transcribed spacers (ITS1 and ITS2), and partial sequences of *18S* and *28S* is deposited in GenBank under the accession number MG652473. This sequence was aligned to other sequences of besnoitiosis agents available in the GenBank database, *B. besnoiti* (DQ227419, DQ227418, AY833646, DQ227420) and *B. caprae* (HM008988), using Muscle in the Mega 7 software [22] (Fig. 5). Sequences of *Toxoplasma gondii* (GenBank: X75429) and *Neospora caninum* (GenBank: GQ899204) were also included as outgroup. Phylogenetic relationships were inferred using three different molecular methods: neighbour joining (NJ), maximum parsimony (MP) and maximum likelihood (ML) with selection of the best model for nucleotide substitution by the Find Best DNA Model test implemented in Mega 7 (for NJ and ML methods). Reliability of the phylogenetic trees using Tamura-3 parameter + I model (NJ and ML) was tested using 1000 bootstrap replicates (NJ, ML and MP).

*Besnoitia bennetti* infection was confirmed by the ITS1 sequencing. The ITS1 sequence (244 bp) was completely identical with *B. bennetti* ITS1 sequences already deposited in the GenBank database (AY827839, AY665399 and JQ013812). ITS1 sequence comparisons between *B. bennetti*, *B. besnoiti*, *B. caprae* and *B. tarandi* have been previously performed [18] and emphasized the close relationship between besnoitiosis agents infecting ungulates [7]. Moreover, as *5.8S* (164 bp), ITS2 (393 bp) and partial *18S* (56 bp) and *28S* (81 bp) sequences are not currently available in the GenBank database for *B. bennetti*, phylogenetic tree reconstructions were performed using this partial 938 bp rDNA sequence with *B. besnoiti* and *B. caprae* rDNA sequences. The phylogenetic tree reconstructed using the NJ method supported an identical topology to that of the ML and MP analyses (Fig. 5). These trees confirmed that *B. bennetti* isolated from the equid host (donkey) was a distinct species to *Besnoitia* species (*B. besnoiti* and *B. caprae*) from bovid hosts (cattle and goat, respectively).

Taking all these results a diagnosis of besnoitiosis was established in both animals.

### Control

A daily treatment based on sulfamethoxazole and trimethoprim (Emdotrim 60% Mix®, 30 mg/kg) was given orally to the affected animal and some improvement was noticed.

### Outcome

Following the diagnosis, the affected animal was treated for four months with sulfamethoxazole and trimethoprim alongside a discontinued hepatoprotective treatment (Sédochol®). The animal gained weight and no more skin conditions were visible.

## Discussion

The current gold standard for diagnosing besnoitiosis in donkeys is histological identification of *Besnoitia* cysts within the dermis of individuals with lesions, generally achieved *via* skin biopsy [15, 23–25]. The size of the cyst and its wall were compatible with *B. bennetti.* Indeed, reported measurement are as follows: 150–450 μm in diameter and 20–50 μm for the cyst wall [18, 23]; however, others [26] reported a thinner cyst wall (< 7 μm). This morphological description is also compatible with *B. besnoiti* in cattle with cysts up to 600 μm in diameter [2, 27]. The nares and the sclera are the most common locations for *Besnoitia* spp. cysts in donkeys [18]; the presence of cysts in the buccal mucosa has been rarely described [28]. Visualizing cysts in two (nares and scleral) or more locations correctly identified 83% of infected donkeys [16]. Serology is less invasive than histological examination and is a better choice for detection of subclinical infection [16]. However, some limitations have been pointed out when it is used alone. Some animals were not detected despite exhibiting severe clinical signs with numerous tissue cysts [16] and it was not possible to discriminate between *B. besnoiti* and *B. bennetti* infection by serology [29].

Ribosomal DNA ITS1 sequences are only available for *B. bennetti* isolated from donkeys in the USA. No ITS1 sequence variation was found between Belgian and USA samples with the same T insertion at position 148 (Additional file 1). The insertion was not present in any other *Besnoitia* species in ungulates (Additional file 1) leading to an identity of 99.7 % with *B. besnoiti*, *B. caprae* and *B. tarandi* [18]. Microsatellites were also used to assess genetic variability between *B. tarandi*, *B. besnoiti* and *B. bennetti* involving intraspecific variations [4, 29, 30]. However, the two *B. bennetti* samples used for these analyses and provided by [17, 18] were previously identified by morphological features and ITS1 sequences [17, 18]. Moreover, in these two studies and for the present work, only one sample of *B. bennetti* was included, preventing investigation of intra-specific variations. To date, no ITS2, *5.8S*, partial and conserved *18S* and *28S* sequences were available for *B. bennetti*. Consequently, this study gives new information concerning these domains, highlighting site variations between *B. bennetti* and *Besnoitia* species infecting other ungulates, leading to identification of a distinct genotype of *B. bennetti*. ITS2 sequence comparison with sequences of *Besnoitia* infecting cattle and goats showed only three different base pairs in the ITS2 domain, two different base pairs in the *5.8S* domain (Additional files 2 and 3) and three to four randomly distributed base exchanges for *18S* and *28S* rDNA, respectively (data not shown). Despite the low level of nucleotide differences between *Besnoitia* agents infecting domestic ungulates, the phylogenetic tree reconstructions showed that *B. bennetti* was not included in the clade involving *Besnoitia* species from cattle and goat with strong supports. This separation was congruent with previous microsatellite studies [4, 29, 30].

Moreover, adding biological features including host range as reviewed by [2] has allowed the collection of evidence to identify *B. bennetti* in this Belgian donkey. The whole genome sequencing of *B. besnoiti* will perhaps provide new molecular targets useful for species phylogeny and genetic population structure.

Concerning haematological evaluation, anaemia was also described but without eosinophilia [23]. However, very little data exists in the literature and these parameters do not seem to be of valuable assistance for diagnosis. Nevertheless, it could be useful for a correct evaluation of renal function before sulfamethoxazole-trimethoprim treatment.

No efficient treatment for besnoitiosis is available. Clinical improvement observed here could be spontaneous and similar to apparent recovery observed in cattle without treatment [3].

In Europe, clinical cases have been reported in France in 1922 [31] and in Spain based on clinical signs and cyst morphology [25]. A very recent serological survey in Spain combining ELISA and western blot confirmation detected antibodies against *Besnoitia* spp. in equids (donkeys, mules and horses) [29]. Unfortunately, neither isolation of parasites nor further molecular genotyping were achieved to determine the accurate *Besnoitia* species involved in these three studies.

This first report of besnoitiosis in donkeys in Belgium suggests an urgent need for an extensive serological survey to assess prevalence in not only Belgian donkey populations but also in France, due to the geographical origin of the mare.

## Conclusions

Difficulties in confirming diagnoses and the absence of efficient treatment options are probably responsible for the underreporting of besnoitiosis in European donkeys. This report aimed to highlight to clinicians the necessity to include besnoitiosis in the differential diagnosis of chronic dermatitis unresponsive to routine topical and systemic treatments. Whether this finding represents an unusual cluster of infections or reflects a wider distribution of subclinical infections which have largely gone undetected to date requires further study. Donkeys are increasing in number and particularly in developing countries [36]. Widespread distribution of this infection would be of international concern and veterinary medical importance. This case report is the first unequivocal report of *B. bennetti* infection of donkey in Europe based on clinical, histological, serological and molecular tools. Further investigations are necessary to unravel the phylogeny of *B. bennetti* and its epidemiology in Europe and elsewhere.

## Additional files

Additional file 1: Alignment of ITS1. Variable sites are highlighted in yellow. (DOCX 116 kb)

Additional file 2: Alignment of ITS2. Variable sites are highlighted in yellow. (DOCX 112 kb)

Additional file 3: Alignment of *5.8S*. Variable sites are highlighted in yellow. (DOCX 66 kb)

## Abbreviations

EFSA: European Food and Safety Authority
ELISA: Enzyme-linked immunosorbent assay
ITS: Internal transcribed spacer
ML: Maximum likelihood
MP: Maximum parsimony
NJ: Neighbour joining
PCR: Polymerase chain reaction
qPCR: Quantitative PCR
rDNA: Ribosomal DNA
