# Supplementary material for: First evidence of Besnoitia bennetti infection (Protozoa: Apicomplexa) in donkeys (Equus asinus) in Belgium
Source: Parasit Vectors. 2018 Jul 18;11:427. doi: 10.1186/s13071-018-2993-3 (PMC6052696; doi:10.1186/s13071-018-2993-3)
Supplement: Supplementary file 3 — Alignment of 5.8S. Variable sites are highlighted in yellow. (DOCX 66 kb) [file 13071_2018_2993_MOESM3_ESM.docx]

**Additional file 3:**

Alignment of 5.8S

| **Species** | **Conutry** | **Domain: 5.8S** | | |  |  |  |  |  |  |  |  |  |  |  |  |  |  |  |  |  |  |  |  |  |  |  |  |  |  |  |  |  |  |  |  |  |  |  |  |  |  |  |
| --- | --- | --- | --- | --- | --- | --- | --- | --- | --- | --- | --- | --- | --- | --- | --- | --- | --- | --- | --- | --- | --- | --- | --- | --- | --- | --- | --- | --- | --- | --- | --- | --- | --- | --- | --- | --- | --- | --- | --- | --- | --- | --- | --- |
| *B. besnoiti* | Israel | DQ227420 | T | A | T | T | T | T | A | A | A | T | T | T | T | C | A | G | C | A | A | T | G | G | A | T | G | T | C | T | T | G | G | C | T | C | G | C | G | C | A | A | [40] |
|  | Spain | DQ227419 | . | . | . | . | . | . | . | . | . | . | . | . | . | . | . | . | . | . | . | . | . | . | . | . | . | . | . | . | . | . | . | . | . | . | . | . | . | . | . | . | [40] |
|  | Spain | DQ227418 | . | . | . | . | . | . | . | . | . | . | . | . | . | . | . | . | . | . | . | . | . | . | . | . | . | . | . | . | . | . | . | . | . | . | . | . | . | . | . | . | [40] |
|  | Portugal | AY833646 | . | . | . | . | . | . | . | . | . | . | . | . | . | . | . | . | . | . | . | . | . | . | . | . | . | . | . | . | . | . | . | . | . | . | . | . | . | . | . | . | [40] |
|  | Spain | EU789637 | . | . | . | . | . | . | . | . | . | . | . | . | . | . | . | . | . | . | . | . | . | . | . | . | . | . | . | . | . | . | . | . | . | . | . | . | . | . | . | . | [40] |
|  | Italy | JF314861 | . | . | . | . | . | . | . | . | . | . | . | . | . | . | . | . | . | . | . | . | . | . | . | . | . | . | . | . | . | . | . | . | . | . | . | . | . | . | . | . | [40] |
|  | Germany | FJ797432 | . | . | . | . | . | . | . | . | . | . | . | . | . | . | . | . | . | . | . | . | . | . | . | . | . | . | . | . | . | . | . | . | . | . | . | . | . | . | . | . | [40] |
| *B. caprae* | Iran | HM008988 | . | . | . | . | . | . | . | . | . | . | . | . | . | . | . | . | . | . | . | . | . | . | . | . | . | . | . | . | . | . | . | . | . | . | . | . | . | . | . | . | [40] |
| *B. bennetti* | Belgium | MG652473 | . | . | . | . | . | . | . | . | . | . | . | . | . | . | . | . | . | . | . | . | . | . | . | . | . | . | . | . | . | . | . | . | . | . | . | . | . | . | . | . | [40] |

| **Species** | **Conutry** | **Domain: 5.8S** | | |  |  |  |  |  |  |  |  |  |  |  |  |  |  |  |  |  |  |  |  |  |  |  |  |  |  |  |  |  |  |  |  |  |  |  |  |  |  |  |
| --- | --- | --- | --- | --- | --- | --- | --- | --- | --- | --- | --- | --- | --- | --- | --- | --- | --- | --- | --- | --- | --- | --- | --- | --- | --- | --- | --- | --- | --- | --- | --- | --- | --- | --- | --- | --- | --- | --- | --- | --- | --- | --- | --- |
| *B. besnoiti* | Israel | DQ227420 | C | G | A | T | G | A | A | G | G | A | C | G | C | A | G | C | G | A | A | A | T | G | C | G | A | **A** | A | C | G | C | A | A | T | G | T | G | A | A | T | T | [80] |
|  | Spain | DQ227419 | . | . | . | . | . | . | . | . | . | . | . | . | . | . | . | . | . | . | . | . | . | . | . | . | . | **.** | . | . | . | . | . | . | . | . | . | . | . | . | . | . | [80] |
|  | Spain | DQ227418 | . | . | . | . | . | . | . | . | . | . | . | . | . | . | . | . | . | . | . | . | . | . | . | . | . | **.** | . | . | . | . | . | . | . | . | . | . | . | . | . | . | [80] |
|  | Portugal | AY833646 | . | . | . | . | . | . | . | . | . | . | . | . | . | . | . | . | . | . | . | . | . | . | . | . | . | **.** | . | . | . | . | . | . | . | . | . | . | . | . | . | . | [80] |
|  | Spain | EU789637 | . | . | . | . | . | . | . | . | . | . | . | . | . | . | . | . | . | . | . | . | . | . | . | . | . | **.** | . | . | . | . | . | . | . | . | . | . | . | . | . | . | [80] |
|  | Italy | JF314861 | . | . | . | . | . | . | . | . | . | . | . | . | . | . | . | . | . | . | . | . | . | . | . | . | . | **.** | . | . | . | . | . | . | . | . | . | . | . | . | . | . | [80] |
|  | Germany | FJ797432 | . | . | . | . | . | . | . | . | . | . | . | . | . | . | . | . | . | . | . | . | . | . | . | . | . | **.** | . | . | . | . | . | . | . | . | . | . | . | . | . | . | [80] |
| *B. caprae* | Iran | HM008988 | . | . | . | . | . | . | . | . | . | . | . | . | . | . | . | . | . | . | . | . | . | . | . | . | . | **.** | . | . | . | . | . | . | . | . | . | . | . | . | . | . | [80] |
| *B. bennetti* | Belgium | MG652473 | . | . | . | . | . | . | . | . | . | . | . | . | . | . | . | . | . | . | . | . | . | . | . | . | . | **G** | . | . | . | . | . | . | . | . | . | . | . | . | . | . | [80] |

| **Species** | **Conutry** | **Domain: 5.8S** | | |  |  |  |  |  |  |  |  |  |  |  |  |  |  |  |  |  |  |  |  |  |  |  |  |  |  |  |  |  |  |  |  |  |  |  |  |  |  |  |
| --- | --- | --- | --- | --- | --- | --- | --- | --- | --- | --- | --- | --- | --- | --- | --- | --- | --- | --- | --- | --- | --- | --- | --- | --- | --- | --- | --- | --- | --- | --- | --- | --- | --- | --- | --- | --- | --- | --- | --- | --- | --- | --- | --- |
| *B. besnoiti* | Israel | DQ227420 | G | C | A | G | A | A | T | T | C | A | G | T | G | A | A | T | C | A | T | C | A | G | A | T | T | T | C | T | G | A | A | C | G | C | A | A | A | T | G | G | [120] |
|  | Spain | DQ227419 | . | . | . | . | . | . | . | . | . | . | . | . | . | . | . | . | . | . | . | . | . | . | . | . | . | . | . | . | . | . | . | . | . | . | . | . | . | . | . | . | [120] |
|  | Spain | DQ227418 | . | . | . | . | . | . | . | . | . | . | . | . | . | . | . | . | . | . | . | . | . | . | . | . | . | . | . | . | . | . | . | . | . | . | . | . | . | . | . | . | [120] |
|  | Portugal | AY833646 | . | . | . | . | . | . | . | . | . | . | . | . | . | . | . | . | . | . | . | . | . | . | . | . | . | . | . | . | . | . | . | . | . | . | . | . | . | . | . | . | [120] |
|  | Spain | EU789637 | . | . | . | . | . | . | . | . | . | . | . | . | . | . | . | . | . | . | . | . | . | . | . | . | . | . | . | . | . | . | . | . | . | . | . | . | . | . | . | . | [120] |
|  | Italy | JF314861 | . | . | . | . | . | . | . | . | . | . | . | . | . | . | . | . | . | . | . | . | . | . | . | . | . | . | . | . | . | . | . | . | . | . | . | . | . | . | . | . | [120] |
|  | Germany | FJ797432 | . | . | . | . | . | . | . | . | . | . | . | . | . | . | . | . | . | . | . | . | . | . | . | . | . | . | . | . | . | . | . | . | . | . | . | . | . | . | . | . | [120] |
| *B. caprae* | Iran | HM008988 | . | . | . | . | . | . | . | . | . | . | . | . | . | . | . | . | . | . | . | . | . | . | . | . | . | . | . | . | . | . | . | . | . | . | . | . | . | . | . | . | [120] |
| *B. bennetti* | Belgium | MG652473 | . | . | . | . | . | . | . | . | . | . | . | . | . | . | . | . | . | . | . | . | . | . | . | . | . | . | . | . | . | . | . | . | . | . | . | . | . | . | . | . | [120] |

| **Species** | **Conutry** | **Domain: 5.8S** | | |  |  |  |  |  |  |  |  |  |  |  |  |  |  |  |  |  |  |  |  |  |  |  |  |  |  |  |  |  |  |  |  |  |  |  |  |  |  |  |
| --- | --- | --- | --- | --- | --- | --- | --- | --- | --- | --- | --- | --- | --- | --- | --- | --- | --- | --- | --- | --- | --- | --- | --- | --- | --- | --- | --- | --- | --- | --- | --- | --- | --- | --- | --- | --- | --- | --- | --- | --- | --- | --- | --- |
| *B. besnoiti* | Israel | DQ227420 | C | A | C | C | A | T | G | G | G | G | A | T | A | C | T | C | T | C | C | T | T | G | G | T | A | C | G | T | C | T | G | **T** | T | T | C | A | G | T | G | T | [160] |
|  | Spain | DQ227419 | . | . | . | . | . | . | . | . | . | . | . | . | . | . | . | . | . | . | . | . | . | . | . | . | . | . | . | . | . | . | . | **.** | . | . | . | . | . | . | . | . | [160] |
|  | Spain | DQ227418 | . | . | . | . | . | . | . | . | . | . | . | . | . | . | . | . | . | . | . | . | . | . | . | . | . | . | . | . | . | . | . | **.** | . | . | . | . | . | . | . | . | [160] |
|  | Portugal | AY833646 | . | . | . | . | . | . | . | . | . | . | . | . | . | . | . | . | . | . | . | . | . | . | . | . | . | . | . | . | . | . | . | **.** | . | . | . | . | . | . | . | . | [160] |
|  | Spain | EU789637 | . | . | . | . | . | . | . | . | . | . | . | . | . | . | . | . | . | . | . | . | . | . | . | . | . | . | . | . | . | . | . | **.** | . | . | . | . | . | . | . | - | [160] |
|  | Italy | JF314861 | . | . | . | . | . | . | . | . | . | . | . | . | . | . | . | . | . | . | . | . | . | . | . | . | . | . | . | . | . | . | . | **-** | - | - | - | - | - | - | - | - | [160] |
|  | Germany | FJ797432 | . | . | . | . | . | . | . | . | . | . | . | . | . | . | . | . | . | . | . | . | . | . | . | . | . | . | . | . | . | . | . | **-** | - | - | - | - | - | - | - | - | [160] |
| *B. caprae* | Iran | HM008988 | . | . | . | . | . | . | . | . | . | . | . | . | . | . | . | . | . | . | . | . | . | . | . | . | . | . | . | . | . | . | . | **.** | . | . | . | . | . | . | . | . | [160] |
| *B. bennetti* | Belgium | MG652473 | . | . | . | . | . | . | . | . | . | . | . | . | . | . | . | . | . | . | . | . | . | . | . | . | . | . | . | . | . | . | . | **C** | . | . | . | . | . | . | . | . | [160] |

| **Species** | **Conutry** | **Domain: 5.8S** | | |  |  |  |  |  |  |  |  |  |  |  |  |  |  |  |  |  |  |  |  |  |  |  |  |  |  |  |  |  |  |  |  |  |  |  |  |  |  |  |
| --- | --- | --- | --- | --- | --- | --- | --- | --- | --- | --- | --- | --- | --- | --- | --- | --- | --- | --- | --- | --- | --- | --- | --- | --- | --- | --- | --- | --- | --- | --- | --- | --- | --- | --- | --- | --- | --- | --- | --- | --- | --- | --- | --- |
| *B. besnoiti* | Israel | DQ227420 | C | T | T | T |  |  |  |  |  |  |  |  |  |  |  |  |  |  |  |  |  |  |  |  |  |  |  |  |  |  |  |  |  |  |  |  |  |  |  |  | [164] |
|  | Spain | DQ227419 | . | . | . | . |  |  |  |  |  |  |  |  |  |  |  |  |  |  |  |  |  |  |  |  |  |  |  |  |  |  |  |  |  |  |  |  |  |  |  |  | [164] |
|  | Spain | DQ227418 | . | . | . | . |  |  |  |  |  |  |  |  |  |  |  |  |  |  |  |  |  |  |  |  |  |  |  |  |  |  |  |  |  |  |  |  |  |  |  |  | [164] |
|  | Portugal | AY833646 | . | . | . | . |  |  |  |  |  |  |  |  |  |  |  |  |  |  |  |  |  |  |  |  |  |  |  |  |  |  |  |  |  |  |  |  |  |  |  |  | [164] |
|  | Spain | EU789637 | - | - | - | - |  |  |  |  |  |  |  |  |  |  |  |  |  |  |  |  |  |  |  |  |  |  |  |  |  |  |  |  |  |  |  |  |  |  |  |  | [164] |
|  | Italy | JF314861 | - | - | - | - |  |  |  |  |  |  |  |  |  |  |  |  |  |  |  |  |  |  |  |  |  |  |  |  |  |  |  |  |  |  |  |  |  |  |  |  | [164] |
|  | Germany | FJ797432 | - | - | - | - |  |  |  |  |  |  |  |  |  |  |  |  |  |  |  |  |  |  |  |  |  |  |  |  |  |  |  |  |  |  |  |  |  |  |  |  | [164] |
| *B. caprae* | Iran | HM008988 | . | . | . | . |  |  |  |  |  |  |  |  |  |  |  |  |  |  |  |  |  |  |  |  |  |  |  |  |  |  |  |  |  |  |  |  |  |  |  |  | [164] |
| *B. bennetti* | Belgium | MG652473 | . | . | . | . |  |  |  |  |  |  |  |  |  |  |  |  |  |  |  |  |  |  |  |  |  |  |  |  |  |  |  |  |  |  |  |  |  |  |  |  | [164] |
